# Estimating the programmatic cost of targeted mass drug administration for malaria in Myanmar

**DOI:** 10.1186/s12889-021-10842-5

**Published:** 2021-04-29

**Authors:** Shwe Sin Kyaw, Gilles Delmas, Tom L. Drake, Olivier Celhay, Wirichada Pan-ngum, Sasithon Pukrittayakamee, Yoel Lubell, Ricardo J. Aguas, Richard James Maude, Lisa J. White, Francois Nosten

**Affiliations:** 1 Department of Tropical Hygiene, Faculty of Tropical Medicine, Mahidol University, Bangkok, Thailand; 2 Mahidol Oxford Tropical Medicine Research Unit, Faculty of Tropical Medicine, Mahidol University, Bangkok, Thailand; 3 Shoklo Malaria Research Unit, Mahidol Oxford Tropical Medicine Research Unit, Mae Sot, Thailand; 4 Department for International Development, London, UK; 5 Centre for Tropical Medicine and Global Health, Nuffield Department of Clinical Medicine, University of Oxford, Oxford, UK; 6 Harvard TH Chan School of Public Health, Harvard University, Boston, USA; 7 Li Ka Shing Centre for Health Information and Discovery, Big Data Institute, University of Oxford, Oxford, UK

**Keywords:** MDA, *P. falciparum*, Malaria elimination, Costs, Myanmar

## Abstract

**Background:**

Mass drug administration (MDA) has received growing interest to accelerate the elimination of multi-drug resistant malaria in the Greater Mekong Subregion. Targeted MDA, sometimes referred to as focal MDA, is the practice of delivering MDA to high incidence subpopulations only, rather than the entire population. The potential effectiveness of delivering targeted MDA was demonstrated in a recent intervention in Kayin State, Myanmar. Policymakers and funders need to know what resources are required if MDA, targeted or otherwise, is to be included in elimination packages beyond existing malaria interventions. This study aims to estimate the programmatic cost and the unit cost of targeted MDA in Kayin State, Myanmar.

**Methods:**

We used financial data from a malaria elimination initiative, conducted in Kayin State, to estimate the programmatic costs of the targeted MDA component using a micro-costing approach. Three activities (community engagement, identification of villages for targeted MDA, and conducting mass treatment in target villages) were evaluated. We then estimated the programmatic costs of implementing targeted MDA to support *P. falciparum* malaria elimination in Kayin State. A costing tool was developed to aid future analyses.

**Results:**

The cost of delivering targeted MDA within an integrated malaria elimination initiative in eastern Kayin State was approximately US$ 910,000. The cost per person reached, distributed among those in targeted and non-targeted villages, for the MDA component was US$ 2.5.

**Conclusion:**

This cost analysis can assist policymakers in determining the resources required to clear malaria parasite reservoirs. The analysis demonstrated the value of using financial data from research activities to predict programmatic implementation costs of targeting MDA to different numbers of target villages.

**Supplementary Information:**

The online version contains supplementary material available at 10.1186/s12889-021-10842-5.

## Background

Countries across the malaria endemic world are aiming to eliminate malaria and committed to identifying approaches aimed at interrupting its transmission [1]. Although substantial progress has been made through the scaling up of existing malaria interventions, the gains achieved are fragile and unevenly distributed among regions and countries. The development of artemisinin resistance in South-East Asia, followed by partner drug resistance [2], led to a call by all country governments in the Greater Mekong Subregion (GMS) to hasten *Plasmodium falciparum* malaria elimination [3].

In 2010, the World Health Organization (WHO) proposed mass drug administration (MDA) as a strategy to accelerate the elimination of multi-drug resistant *P. falciparum* malaria [4]. This strategy aims to treat every individual in a community with three rounds of a full dose of antimalarial drug, regardless of whether they have malaria symptoms. Many field trials and programmatic implementations of MDA have been carried out over the past century with varying degrees of success [5]. However, a trial MDA conducted in Zanzibar showed no impact on malaria incidence [6].

Targeted MDA, sometimes referred to as focal MDA [7, 8], is the practice of delivering MDA to high incidence subpopulations only, rather than the entire population. A series of targeted MDA projects have recently been piloted in countries in the GMS [9–12] and Africa [13, 14]. These trials have demonstrated that MDA is feasible and well-accepted by communities, with high levels of community participation. MDA using a therapeutic dose of an effective antimalarial medication can clear the malaria parasite reservoir, including asymptomatic infections that would otherwise not be treated [15]. This approach can rapidly reduce malaria parasite prevalence if highly efficacious antimalarial drugs are used [16].

The Malaria Elimination Task Force (METF), established by the Shoklo Malaria Research Unit, which is based on the Thai–Myanmar border, delivered an integrated malaria elimination strategy that layered targeted MDA over a series of malaria control and research activities in the region. Malaria transmission is low, seasonal, and spatially heterogeneous in Kayin State, Myanmar [17]. Five decades ago, malaria was a significant problem, but following rigorous treatment interventions and implementation of malaria posts by the METF, the annual incidence of malaria has gradually decreased in the region. Malaria posts are operated by trained members of the community. Malaria infections, confirmed at malaria posts using a rapid diagnostic test (RDT), comprise approximately 12% of all febrile illness [15]. In Myanmar, malaria infection is frequently asymptomatic [18]. A recent survey using ultrasensitive polymerase chain reaction (uPCR) showed a malaria prevalence of 21%, with *P. falciparum* and *P. vivax* comprising 3 and 15% of infections, respectively [15]. MDA was conducted in 61 selected villages in five phases over 2 years, with more than 80% participation by village residents. As a result, the incidence of *P. falciparum* malaria was reduced by 92%. Following targeted MDA in the region, 71% of METF villages reported no *P. falciparum* malaria [19].

In addition to the effectiveness of interventions, policymakers must be able to evaluate the costs of malaria elimination, to determine which intervention or package of interventions should be invested in, given the constraints of budget limitations. Even when the epidemiological impact of intervention packages is well characterised, efficient resource-allocation decisions can only be made when all resources consumed are explicitly valued and visible. Cost analyses have been conducted for most malaria control activities [20], but to date, few costing exercises of MDA have been performed [21].

Here, we used financial data from METF targeted MDAs in 61 selected villages in Kayin State, eastern Myanmar, as the basis for a costing exercise to analyse the costs of delivering MDA in targeted villages in Myanmar and calculate the scalable cost of delivering targeted MDA to support *P. falciparum* malaria elimination. A micro-costing approach allows for precise assessment of the true costs of health interventions. The costing involves the direct enumeration and costing out of every item considered to be a resource utilised in a particular process of interest i.e. integrated malaria elimination strategies. This approach has been used to estimate of malaria community health workers in Myanmar [22] and MDA in African countries [23].

## Methods

### Study design

This study was a retrospective cost analysis to estimate the programmatic cost of targeted MDA in Myanmar. In Myanmar, targeted MDA was implemented in three sites: 10 villages in Phayarthonesu sub-township, 3 villages in southern Myanmar and 61 villages in Kayin State in eastern Myanmar. In this cost analysis, we explored the programmatic cost of targeted MDA implemented by METF in Kayin State, which covers 365,000 people.

### Study area

Kayin State lies on Myanmar’s international border, with Thailand to the east. It is a mountainous region, with the rocky Dawna mountain range running the length of eastern Kayin State. The climate is hot and humid, with average maximum temperatures of between 29 and 37 °C and average annual rainfall of approximately 5000 mm [24]. The METF covers a population of 365,000 people. The residents of Kayin State traditionally rely on agriculture for their livelihoods; major crops include rice, rubber, sugarcane, coffee, and seasonal fruit and vegetables.

Kayin State has experienced decades of armed conflict between various Kayin militant groups and the national government [25]. Therefore, it is a politically sensitive area, and government accessibility to the region is limited. Consequently, basic infrastructure, such as roads, electricity, schools, and health care facilities, are under-developed.

### Data collection

A series of consultations were held with METF, and the importance of the costing exercise was explained. Permission to access financial reports relating to the targeted MDA conducted by METF (2015) was obtained and all information required for the costing analysis was collected.

### Costing

A micro-costing approach was used to estimate the costs of activities necessary to conduct a targeted MDA in four townships in Kayin State (Myawaddy, Hpapun, Hlaingbwe, and Kawkareik). This approach is particularly useful for estimating the unit delivery costs of community-based interventions or treatments and new technologies. It enables the accurate assessment of health interventions by collecting details on resources used and respective unit costs.

### Data analysis

The financial data were entered into an Excel spreadsheet then transferred and analysed in R software version 3.6.3. A descriptive statistical analysis was performed and the resources used in the activities that comprise targeted MDA were calculated.

### Cost model

A cost model was developed to estimate the implementation cost of all activities related to targeted MDA in four townships in Kayin State. The costs of implementing targeted MDA include (i) community engagement, (ii) identification of villages for MDA via uPCR surveys, and (iii) conducting mass treatment in target villages. METF rolled-out their targeted MDA campaigns in phases. The malaria elimination initiative was delivered to 1226 villages, of which 30 received targeted MDA in 2015. First, we estimated the cost of implementing MDA during 2015 based on 2015 financial data; these costs were then extrapolated to a total of 61 villages that received targeted MDA.

All resource ingredients required to perform the three MDA implementation activities were identified, measured, and valued by reviewing financial reports and conducting interviews with key staff. Then, each cost was assigned to a primary resource cost centre. Resource costs were estimated by multiplying the unit cost by the quantity of the resource used. Costs in resource centres were re-classified into relevant activity cost centres. Then, the total cost of each activity and the cost per person reached were estimated. The cost per person reached was calculated by the total cost (targeted MDA after community engagement and the village-level prevalence survey) divided by the total population in that area (365000). These targeted MDA costs were shared among all people in the region because targeted MDA was provided in addition to other malaria interventions. A detailed breakdown of the costs can be found in the online tool.

### Cost model ingredients

i) Staff costs. These comprised basic salaries for both national and international staff, plus their allowances, which included benefits and overtime. These staff costs were shared resources, so the allocation of these shared resources was based on the proportion of the time these staff contributed to various services.
ii) Travel costs. These included all transportation costs, including bus, taxi, and boat fares; toll fees; petroleum consumed during the project; motorbikes and their maintenance; and other travel-related expenses, such as accommodation and *per diems*. The cost of transportation of medical and non-medical products and travel expenses for monitoring and training were also included in the travel cost centre.
iii) Consumables costs. These included the cost of media, pamphlets, and other health education materials for community engagement activities; uPCR sample preparation and analysis; antimalarial drugs; pharmacovigilance costs and medication to treat any adverse effects. The costs of consumables used for uPCR sample collection and analysis were estimated based on the mean number of uPCR samples and the unit cost of sample collection.
iv) Overheads. These consisted of central and field office rental fees, utility bills, office computers and software, and office furniture. These costs were annualised over the years they were expected to be used to estimate annual equivalent values. The useful life of traded capital goods was taken from WHO-CHOICE (https://www.who.int/choice/cost-effectiveness/inputs/capital_goods/en/).
v) Incentives. These comprised community incentives, e.g. the cost of large water containers for a community or the cost of constructing sanitary pit latrines in the common areas of villages targeted for MDA. Participants were also provided with snacks and drinks during the MDA campaign and after completing MDA.

All costs of resources consumed during the MDA campaign were initially analysed in the local currency and then converted to United States dollars (US$), based on historical exchange rates from the Forex website. The median exchange rate in 2015 (1 US$ = 33.14 Thai Baht) was used for the currency conversion.

### Sensitivity analysis

While models can deliver a single summary outcome, the interpretation of the results depends on the level of confidence or uncertainty. One-way sensitivity analyses were conducted to investigate the robustness of the estimated results by running the cost model and varying the assumptions of the key parameters one at a time while the rest of parameters were kept constant. The results of the sensitivity analysis are presented in a tornado diagram [26].

### Online tool

The cost model is available online: https://moru.shinyapps.io/Mass-Malaria-Interventions-Costing-Tool/. Details of the model’s construction and parameters used are given in the supplementary information (Additional file 1 .doc).

## Results

**Fig. 1:**
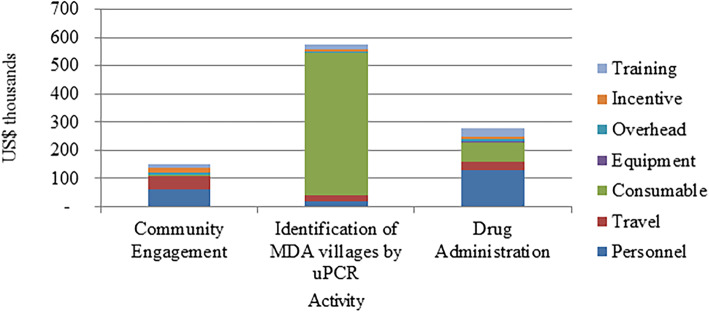
The total cost of targeted mass drug administration in Kayin State, Myanmar, based on financial data provided by the Malaria Elimination Task Force. MDA, mass drug administration

The METF implemented an integrated malaria elimination initiative in 1226 villages in Kayin State from May 2014 to December 2019. This included targeted MDA in 61 villages in areas with high levels of sub-microscopic *P. falciparum* malaria, to contain further spread of multi-drug resistant malaria. The total cost of the targeted MDA was estimated using a micro-costing approach. Table 1 and Fig. 1 show the detailed breakdown of the total cost of targeted MDA in Kayin State, which involved three activities: i) community engagement (CE), ii) identification of target villages based on uPCR results, and iii) targeted mass treatment in selected villages. The total cost of CE activity for 61 villages (5% of 1226 villages) was US$ 76,330. The average cost per person for providing CE was US$ 0.20.

**Table 1:** The cost breakdown of the total programmatic cost of targeted MDA in Kayin State, Myanmar

	Consumables	Equipment	Incentives	Programme	Personnel	Training	Travel	Total costs
Community engagement	1116	0	18,018	8544	27,718	9308	11,625	76,330
Identification of targeted MDA villages	435,456	2700	19,440	5126	34,656	9464	34,200	541,042
Mass treatment	60,853	4650	26,040	8544	135,159	28,313	28,200	291,759
**Total costs**	**497,425**	**7350**	**63,498**	**22,214**	**197,533**	**470 85**	**74,025**	**909,131**

*MDA* Mass drug administration

Villages for targeted MDA were identified based on their malaria prevalence relative to other villages in Kayin State. A malaria prevalence survey was conducted, using a uPCR assay, to measure the true prevalence of malaria in 272 randomly selected villages. Consumables comprised the largest proportion of the total cost of identifying targeted MDA villages, accounting for 80% of all costs. This was because of the cost of uPCR analysis, which was approximately US$ 25 per test. The high cost of the tests was due to the need for expensive equipment, reagents and consumables for high-volume PCR to obtain the desired sensitivity [27]. The total cost of the prevalence survey for 272 villages was US$ 541,042, with the average cost per village of US$ 1989.

The cost of providing three rounds of three-day antimalarial mass treatment in 61 villages was US$ 291,759. The staff costs were the largest contributor to the total cost of mass treatment (46%), followed by consumables (21%). This was because the METF provided supervised treatment for every dose of antimalarial treatment. Therefore, staff needed to stay for at least 4 days in a village in each treatment round to cover any latecomers and to watch for any side effects of the treatment. The average cost per village for providing a full course (three-day treatment) of antimalarial drugs for three consecutive months was US$ 4455. The cost per person reach for providing three rounds of antimalarial mass treatment was US$ 0.8.

The total cost of an integrated MDA initiative, including detection of hotspots and mass treatment in 61 villages (5% of villages), would be approximately US$ 910,000 over 2 years. The cost per capita for three rounds of targeted MDA was estimated to be US$ 2.5. The intervention was an integrated strategy for the prevention, early detection, and treatment of clinical malaria in all villages, combined with MDA in targeted villages; therefore, the cost per capita quoted is derived from the total cost of the integrated MDA package of interventions but excluding early detection and case management divided by the total population of the area where it was delivered (including MDA villages and non-MDA villages). This figure allows for comparison between scenarios where different targeting strategies and different populations are considered.

**Fig. 2:**
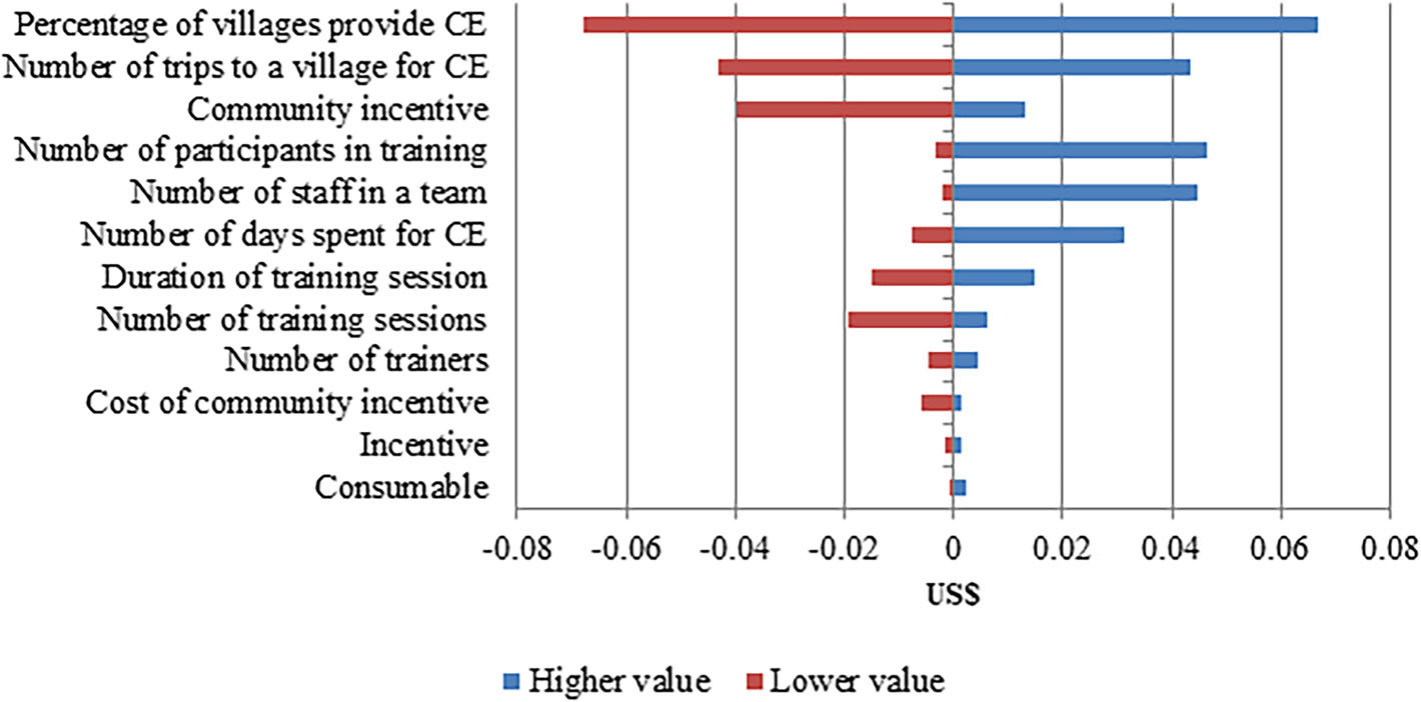
The variation in the cost of community engagement due to changes in a range of model parameters. CE, Community Engagement

**Fig. 3:**
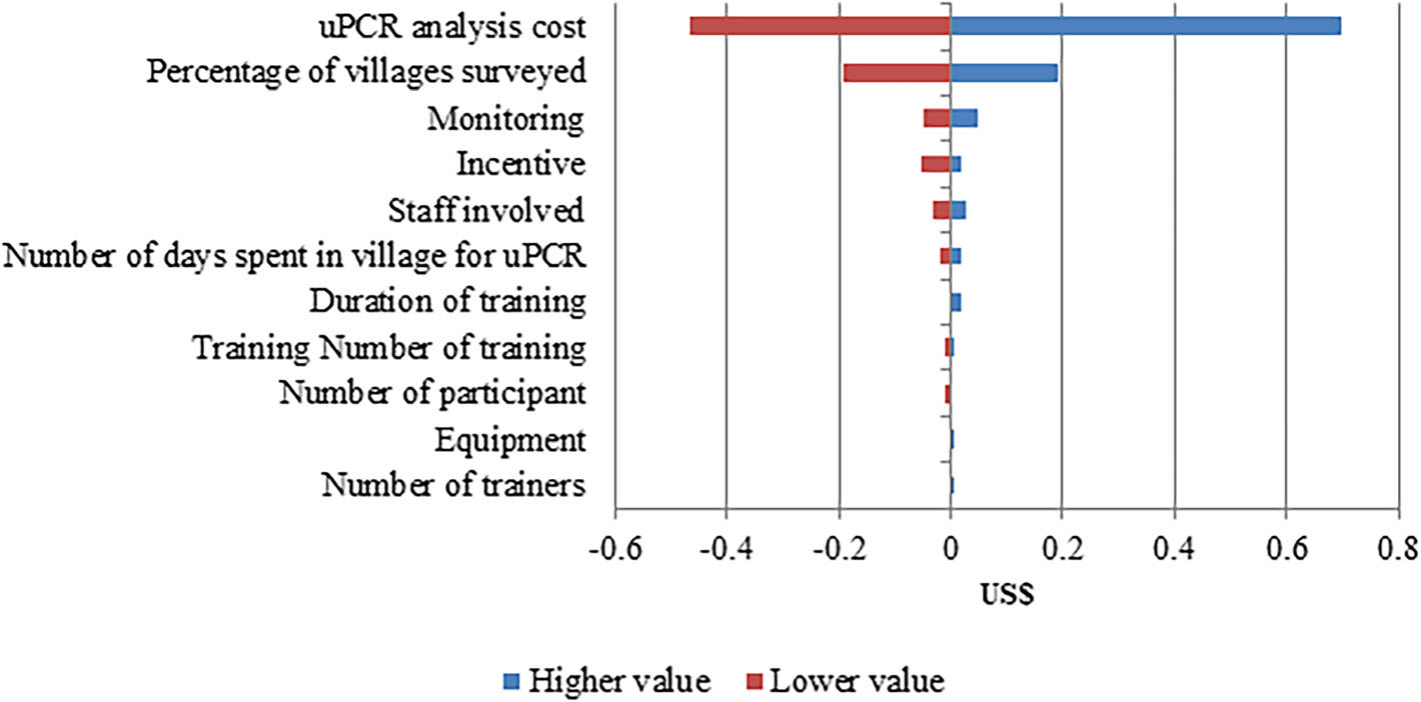
The variation in the cost of identification of villages for MDA due to changes in a range of model parameters. uPCR, ultrasensitive polymerase chain reaction

**Fig. 4:**
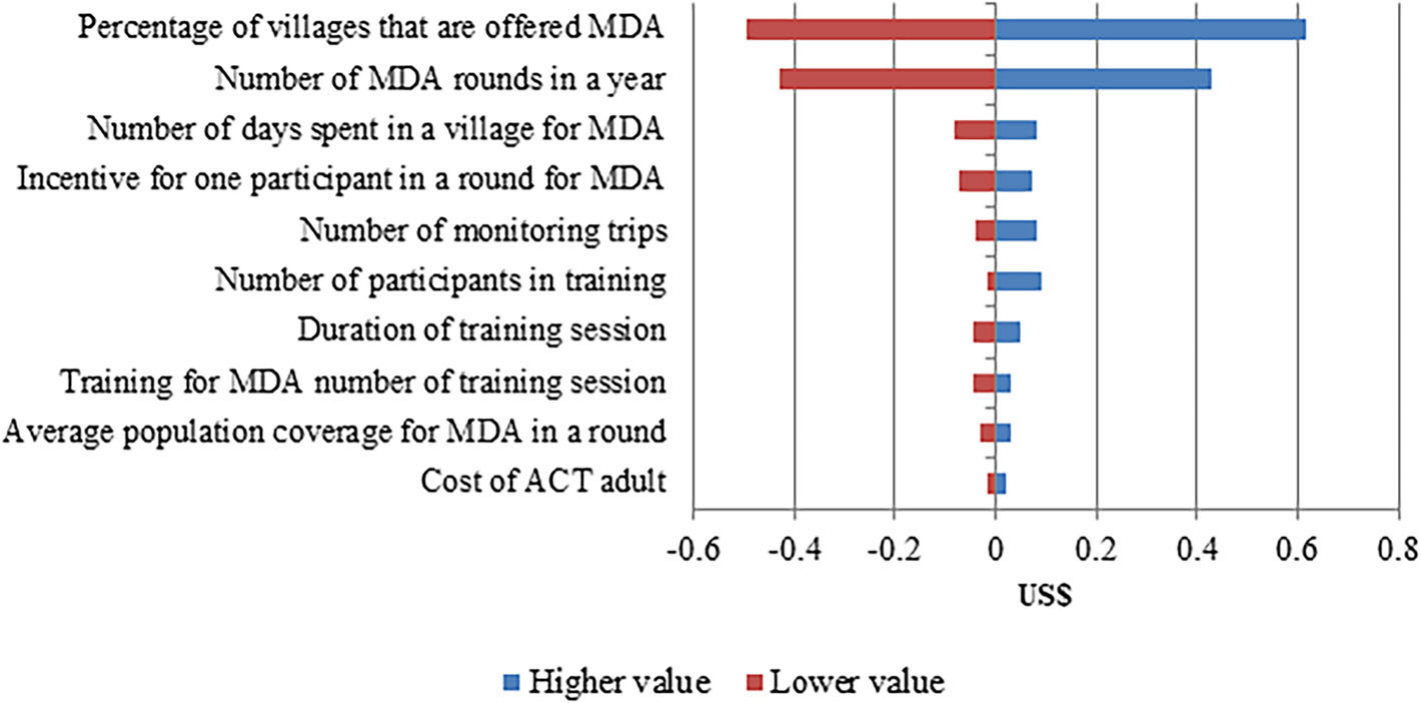
The variation in the cost of targeted mass treatment due to changes in a range of model parameters. MDA, mass drug administration; ACT, Artemisinin Combination Therapy

Figures 2, 3 and 4 show the sensitivity analyses of the activities of targeted MDA using the parameter values shown in Table 2. The cost model to estimate the cost of CE was sensitive to the parameters for percentage of villages provided with CE, the number of trips to a village for CE activity and the cost of community incentives. The cost of identifying villages for targeted MDA was most sensitive to the cost of uPCR analysis and the percentage of villages to perform survey. The percentage of villages offered targeted MDA and the number of MDA rounds were the biggest contributors to the estimated costs of mass treatment.

**Table 2:** Model parameter ranges for the sensitivity analysis

**Community engagement**	**Default**	**Maximum**	**Minimum**	**Comments**
Number of days spent in a village for CE	1	3	0.5	METF trial
Number of staff in a team	3	8	2	METF trial
Consumables	18	20.7	15.3	+/−15%
Refreshments	19	21.85	16.15	+/−15%
Number of villages provided with a community incentive	3	4	0	Assumption
Cost of community incentives	4828	5000	4103.8	+/−15%
Percentage of villages for CE	5%	10%	0	Assumption
Number of training sessions	4	5	1	Assumption
Number of participants in one training session	10	40	8	Assumption
Number of trainers in one training session	2	3	1	Assumption
Duration of training session	3	5	1	METF trial
Number of trips to a village for community engagement	3	5	1	METF trial
**Identification of targeted MDA villages**	**Default**	**Maximum**	**Minimum**	**Comments**
Percentage of villages surveyed to identify hotspot villages	22	25.3	18.7	+/− 15%
Number of days spent in a village for uPCR	1	1.5	0.5	METF trial
Staff involved	4	5	2	METF trial
Incentive for one participant	1	2	0	METF trial
Equipment	5	9.25	4.25	+/−15%
Consumables	1	1.15	0.85	+/− 15%
uPCR analysis cost	25	40	15	METF trial
Number of training sessions	6	7	3	Assumption
Number of participants	20	20	10	Assumption
Number of trainers	2	3	1	Assumption
Duration of training sessions	1	2	0.5	METF trial
Monitoring trip	1	2	0	Assumption
Number of days spent in a village for monitoring	5	10	2	Assumption
**Mass treatment**	**Default**	**Maximum**	**Minimum**	**Comments**
Percentage of villages that are offered targeted MDA activity	5%	10%	1%	Assumption
Number of targeted MDA rounds in a year	3	5	1	Assumption
Average percentage of population coverage for targeted MDA in a round	70%	80.5%	59.5%	+/− 15%
Number of days spent in a village for targeted MDA activity	7	9	5	METF trial
Incentive for one participant in a round for targeted MDA	1	2	0	Assumption
Number of trips requiring car rental during targeted MDA activity	15	15	8	METF trial
Equipment cost per village	25	28.75	21.25	+/− 15%
Consumables cost per village	11	12.65	9.35	+/− 15%
Cost of ACT child	0.93	1.0695	0.7905	+/− 15%
Cost of ACT youth	1.46	1.679	1.241	+/−15%
Cost of ACT adult	1.98	2.277	1.683	+/−15%
Cost of primaquine	0.01	0.0115	0.0085	+/− 15%
Cost of medicine for treatment of side-effects per village	39	44.85	33.15	+/− 15%
Antimalarial drug wastage	5	10	3	Assumption
Number of training sessions	5	7	2	Assumption
Number of participants	20	50	15	Assumption
Number of trainers	3	5	2	Assumption
Duration of training sessions	3	5	1	METF trial
Number of monitoring trips	1	3	0	Assumption
Duration of monitoring trips	10	15	5	Assumption

*MDA* Mass drug administration; *uPCR* Ultrasensitive polymerase chain reaction; *ACT* Artemisinin Combination Therapy; *METF* Malaria Elimination Task Force

### Estimating the programmatic cost of targeted MDA to support *P. falciparum* malaria elimination in Kayin State

The METF screened 272 villages (22% of all villages) to determine malaria prevalence and then performed targeted MDA in 61 selected villages (5% of all villages) based on the survey results. The cost of targeted MDA is highly dependent on the number of target villages. The more villages targeted for MDA, the faster the decline in prevalence in the whole area (assuming all targeted MDA can be implemented in a reasonable amount of time, as MDA is a time-limited process), but the greater the resources need to be invested. The cost of conducting the prevalence survey for 272 villages was kept constant, and we then estimated the programmatic cost of targeted MDA for different proportions of target villages. The detailed breakdown of the cost of targeted MDA, depending on the percentage of villages targeted for MDA, is shown in Table 3.

**Table 3:** Two-year programmatic costs of targeted MDA with different numbers of villages selected for targeted MDA

Cost of screening 272 villages using uPCR	Percentage of villages with targeted MDA	Number of villages	Cost of community engagement	Cost of mass treatment	Total cost	Cost per village^a^	Cost per person reached^b^
541,042	1%	13	56,735	111,551	709,328	579	1.9
541,042	2%	25	61,534	155,684	758,260	618	2.1
541,042	3%	37	66,332	199,816	807,190	658	2.2
541,042	4%	50	71,531	247,626	860,199	702	2.4
541,042	5%	62	76,330	291,759	909,131	742	2.5
541,042	6%	74	81,128	335,891	958,061	781	2.6
541,042	7%	86	85,927	380,024	1,006,993	821	2.8
541,042	8%	99	91,126	427,834	1,060,002	865	2.9
541,042	9%	111	95,924	471,966	1,108,932	905	3.0
541,042	10%	123	100,732	516,099	1,157,864	944	3.2

^a^Cost per village is estimated by dividing the total cost of targeted MDA by the total number of villages in the four townships (1226 villages). These targeted MDA costs will be shared among all villages in the region because targeted MDA is provided in addition to other malaria interventions, so the total cost is distributed among all villages in the region

^b^Cost per person reached is calculated by the total cost divided by the total population in that area (365000)

*MDA* Mass drug administration; *uPCR* Ultrasensitive polymerase chain reaction

For example, if 10% of villages are assumed to be provided with MDA, the total programmatic cost of targeted MDA would be approximately US$ 1.15 million. The average cost per village and the average cost per person reached would be US$ 944 and US$ 3.2, respectively in this example.

### Comparing the programmatic cost of targeted MDA using different infection diagnostic approaches

We estimated the programmatic cost of targeted MDA using different molecular diagnosis methods, such as uPCR, RNA testing and ELISA testing. The unit cost of molecular tests varies, so the programmatic cost of targeted MDA in Kayin State will differ if we use techniques other than the more expensive uPCR method. The percentage of the prevalence survey was kept the same as the METF project (22% of all villages); only the percentage of villages where CE and mass treatment were provided was varied. Table 4 shows the comparison of the programmatic cost of targeted MDA using three different molecular diagnosis methods. The unit costs set for the RNA and ELISA tests per sample were US$ 20 and US$ 5, respectively (F. Nosten, personal communication). The total programmatic cost would be decreased by approximately 37% if METF used the ELISA method to identify villages to target for MDA. Similarly, the cost of targeted MDA would be reduced by 9% if uPCR was switched to RNA testing.

**Table 4:**
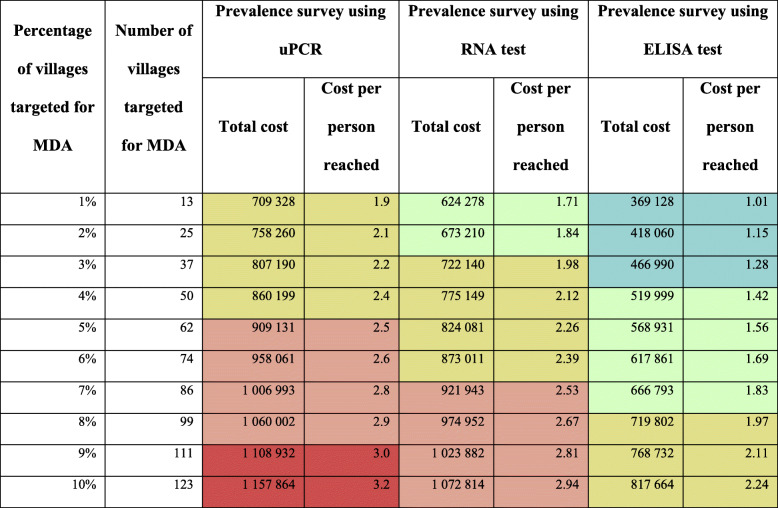
Detailed costs of targeted MDA using three different molecular assays to identify hotspot villages. Cells are highlighted with different colours to illustrate the areas of equivalent costs in the three strategies

*MDA* Mass drug administration; *uPCR* Ultrasensitive polymerase chain reaction

## Discussion

The malaria map is shrinking in both Kayin State [19] and the whole of Myanmar [28]. Myanmar has set a goal to interrupt transmission and eliminate *P. falciparum* malaria from the entire country by 2025 [29]. Malaria elimination requires a substantial level of investment, especially for detecting and responding to small numbers of remaining malaria cases [30]. Which malaria intervention packages to use and the resources needed to eliminate this disease nationally and sub-nationally is a challenging question for a developing country that largely relies on external funding to achieve this goal.

Malaria case management and intensive vector control are core interventions in malaria control, but achieving elimination goals is likely to require other, population-wide measures, particularly in the context of increasing multi-drug resistant malaria. A population-wide, medicine-based strategy, such as MDA, can accelerate the reduction in transmission [31]. Several targeted MDA projects have been conducted in the GMS, including in Kayin State [15], and have been shown to reduce the incidence and prevalence of malaria [32]. The implementation of targeted MDA, however, requires a significant investment in terms of resources and time to mobilise the targeted villages. The higher cost relative to standard approaches to malaria control and elimination was considered acceptable given the risk of multi-drug resistance and the measures deemed necessary to address this.

The cost of identifying target villages was the largest contributor in this cost analysis. When the prevalence of malaria is declining, its management is focused on subclinical infection. In low transmission settings, asymptomatic infection dynamics should be adequately identified using highly sensitive diagnostic methods. Molecular techniques are more sensitive than other diagnostic methods. The detection limit of PCR is approximately 22 parasites per mL. METF used high-volume uPCR to identify villages to target for MDA. An alternative assay to uPCR, such as RNA or ELISA, would reduce the cost of the prevalence survey while maintaining sufficient sensitivity [33].

Studies on the cost of deploying malaria MDA are limited, with one study examining the delivery costs of MDA in two island settings and an emergency setting [21]. The average cost per person reached for three rounds of MDA conducted in Comoros, Vanuatu and Sierra Leone were estimated at US$ 42.39, 17.85 and 3.93, respectively. These costs were higher than our estimated cost per person reached for three rounds of targeted MDA. A recently published article evaluated the cost-effectiveness of focal MDA and MDA in Zambia [34] and estimated the cost per person reached for MDA to be US$ 9.42, which was also higher than our estimate costs of targeted MDA.

Although the METF study was not designed to assess non-targeted MDA, we calculated the cost of a hypothetical non-targeted MDA in our setting. The MDA costs for the targeted villages were selected from Table 3, while excluding the costs of targeting using uPCR. The per capita cost was estimated using the population of only those villages receiving MDA, estimated based on an average village size of 200. This led to an estimate of between US$ 25 and 65 per capita.

The sensitivity analysis was carried out where possible with lower and upper limits for each input parameter informed by realistic operational limitations of the METF study (Table 2), making some of the output asymmetrical around the default value. Where such information was not available, the limits used were ± 15% of the default values. The cost was most sensitive to the percentage of all villages that were provided with CE. More villages were provided CE in Kayin state, this led to higher cost per capita for that region. The number of visits to the village also contributed to the cost of CE. The CE team organized many CE sessions to build relations and trust with the villagers. Sometimes, the staff had to pay additional visits to understand the residents’ concerns and facilitate community ownership of the malaria elimination project. For the community incentive (also a high sensitivity parameter), if the program manager decided not to provide community incentive (e.g., constructing a big water tank for the community) the cost per capita for CE will be reduced by US$ 0.04; therefore, the average cost per person for providing CE was US$ 0.16. The number of participants in the training can vary up to a (operationally feasible) maximum, thus increasing the cost per capita for CE. Similarly, increasing the size of the team considerably impact to the cost per capita for CE. The cost per capita for survey is highly sensitive to the uPCR analysis cost and the proportion of villages surveyed for malaria prevalence estimates. Similarly, the higher number of villages offered MDA in the region, the greater the cost per capita for MDA.

In South-East Asia, only two countries have been declared malaria-free, the Maldives (2015) and Sri Lanka (2016). Both are likely to have benefitted from their geographical isolation. Looking back on the success story in Sri Lanka, it took decades of effort with a multidimensional approach that included combined vector control, case management and disease surveillance. A genomic epidemiology study that collected data from 2008 to 2018 [35] revealed that the spread of multi-drug resistant *P. falciparum* malaria in GMS countries was accelerating, highlighting the urgent need to adopt an effective strategy to eliminate malaria. Recently published studies [11, 15, 32, 36] suggest that targeted/focal MDA with a high degree of community participation can rapidly reduce malaria infections to zero when used in conjunction with intensive vector control and standard case management. There was no significant increase in any of the genetic markers for resistance after MDA [32]. The components necessary for a successful integrated malaria elimination strategy are predicted to be highly dependent on the setting [37], and Myanmar is expected to require MDA or other more intensive interventions.

Policymakers must therefore consider a trade-off between investing in rapid elimination strategies that might stave off the threat of resurging drug resistant malaria, or slower (and cheaper) elimination strategies. GMS countries need to buy time to halt the spread of multi-drug resistant malaria while new antimalarial drugs are developed. This analysis provides the added cost of targeted MDA to rapidly eliminate malaria on top of existing malaria surveillance and control costs. We estimated the programmatic cost of targeted MDA in Kayin State using financial data from the METF implementation, and developed a malaria mass intervention costing tool to support policy decisions towards *P. falciparum* malaria elimination in other settings. The key features of this costing tool are its ease of use, the flexibility to explore different targeting strategies, and the cost predictions for any single malaria intervention or package of interventions.

The costing tool was designed based on the targeted MDA initiative in Myanmar; nonetheless, the tool can also be used to predict programmatic costs in other GMS countries, by adjusting the unit costs of resources and the proportion of villages undergoing interventions. For example, the tool could be deployed in other regions in Myanmar, such as Chin State, where *P. falciparum* malaria incidence and mortality is high in comparison with other regions [28]. The added benefit, beyond addressing the multi-drug resistance issue, would be the additional lives saved by accelerating elimination to a date earlier than 2030. Table 3 allows the exploration of the cost implications of delivering this programme in a higher prevalence setting, which would probably mean a higher number of villages fulfilling the conditions for being designated a hotspot. Table 4 allows the exploration of the trade-off between costs and the use of cheaper alternative screening options.

There are several limitations to this cost analysis. Different teams providing MDA include staff members of differing levels of seniority, so there may be some variations in estimating staff costs. However, this variation between MDA teams is negligible. As the percentage of villages provided with MDA increases, programme managers have the option of training more staff or using their existing team for an extended period. In our cost estimation, we used the same team to provide MDA. Cost variations may result if a programme manager makes the trade-off of recruiting more staff to complete MDA in less time. The targeted MDA initiative in this cost analysis was operated by a Thailand-based organisation. Therefore, staff compensation and travel costs to access the villages were based on staff travelling from the Thai side, so some cost variations will be seen if villages were accessed from the Myanmar side. However, this variation would be minimal, since most of the costs were incurred within the country. This cost analysis included the costs incurred in the field but did not include the costs of strategic meetings with higher level government officers. The reason for this is that the program manager visited two times to the central Disease Control Unit and the cost in total for both meetings (including ticket, visa and hotel accommodation) was US$ 1093. This cost distributed across the study population giving a cost per capita of US$ 0.003 which is minimal. When applying this ingredients-based approach to future interventions with more people involved in such meetings and at a higher frequency, then this cost should be included.

Our costing model can predict the costs of a particular malaria elimination package design, but cannot make any assurances on the likelihood of success of such a package in achieving elimination elsewhere. It is designed to be used in concert with detailed knowledge of the target area and/or with mathematical models that can simulate the impact of various strategy designs on the prevalence and incidence of malaria [37].

## Conclusions

This cost analysis quantifies the costs of accelerating *P. falciparum* malaria elimination. Such cost analysis makes a useful contribution to determine the level of resources required to clear the residual malaria parasite reservoir. It also provides a framework for projecting the cost of similar programmes in settings with different epidemiology and/or the exploration of the cost of alternative designs. The study demonstrated the use of financial data from MDA research to project the programmatic implementation cost of MDA with a different number of targeted villages.

## Supplementary Information

**Additional file 1.** The interactive costing tool. This file contains the features of malaria mass intervention costing tool.

## Data Availability

The datasets used and/or analysed during this cost analysis are available from the corresponding author on reasonable request. Public access to the database is not available. The author obtained administrative permission to access the data and used these in the Ethics approval and consent to participate section.

## Abbreviations

MDA: Mass drug administration
GMS: Greater mekong subregion
WHO: World health Organization
METF: Malaria elimination task force
uPCR: Ultrasensitive polymerase chain reaction
US$: United States dollars
RDT: Rapid diagnostic test
CE: Community engagement
ELISA: Enzyme-linked immunosorbent assay
CHW: Community health worker
DHA: Dihydroartemisinin
PQP: Piperaquine phosphate

## References

[1] World Health Organisation: Global Technical Strategy for Malaria, 2016–2030. 2015.

[2] Ashley EA, Dhorda M, Fairhurst RM, Amaratunga C, Lim P, Suon S, Sreng S, Anderson JM, Mao S, Sam B, Sopha C, Chuor CM, Nguon C, Sovannaroth S, Pukrittayakamee S, Jittamala P, Chotivanich K, Chutasmit K, Suchatsoonthorn C, Runcharoen R, Hien TT, Thuy-Nhien NT, Thanh NV, Phu NH, Htut Y, Han KT, Aye KH, Mokuolu OA, Olaosebikan RR, Folaranmi OO, Mayxay M, Khanthavong M, Hongvanthong B, Newton PN, Onyamboko MA, Fanello CI, Tshefu AK, Mishra N, Valecha N, Phyo AP, Nosten F, Yi P, Tripura R, Borrmann S, Bashraheil M, Peshu J, Faiz MA, Ghose A, Hossain MA, Samad R, Rahman MR, Hasan MM, Islam A, Miotto O, Amato R, MacInnis B, Stalker J, Kwiatkowski DP, Bozdech Z, Jeeyapant A, Cheah PY, Sakulthaew T, Chalk J, Intharabut B, Silamut K, Lee SJ, Vihokhern B, Kunasol C, Imwong M, Tarning J, Taylor WJ, Yeung S, Woodrow CJ, Flegg JA, Das D, Smith J, Venkatesan M, Plowe CV, Stepniewska K, Guerin PJ, Dondorp AM, Day NP, White NJ, Tracking Resistance to Artemisinin Collaboration (TRAC) (2014). Spread of artemisinin resistance in plasmodium falciparum malaria. N Engl J Med.

[3] Ministry of Health and Sports Myanmar. Six Mekong Nations call for accelerated action to eliminate malaria before 2030. 2018. https://www.mohs.gov.mm/Main/content/new/six-mekong-nations-call-for-accelerated-action-to-eliminate-malaria-before-2030.

[4] World Health Organisation. Consideration of Mass Drug Administration for the containment of artemisinin-resistant malaria in the Greater Mekong Subregion. 2010.

[5] Newby G, Hwang J, Koita K, Chen I, Greenwood B, von Seidlein L, Shanks GD, Slutsker L, Kachur SP, Wegbreit J, Ippolito MM, Poirot E, Gosling R (2015). Review of mass drug administration for malaria and its operational challenges. Am J Trop Med Hyg.

[6] Morris U, Msellem MI, Mkali H, Islam A, Aydin-Schmidt B, Jovel I, Shija SJ, Khamis M, Ali SM, Hodzic L, Magnusson E, Poirot E, Bennett A, Sachs MC, Tarning J, Mårtensson A, Ali AS, Björkman A (2018). A cluster randomised controlled trial of two rounds of mass drug administration in Zanzibar, a malaria pre-elimination setting-high coverage and safety, but no significant impact on transmission. BMC Med.

[7] Eisele TP, Silumbe K, Finn T, Chalwe V, Kamuliwo M, Hamainza B (2015). Assessing the effectiveness of household-level focal mass drug administration and community-wide mass drug administration for reducing malaria parasite infection prevalence and incidence in Southern Province, Zambia: study protocol for a community randomized controlled trial. Trials.

[8] Hsiang MS, Ntuku H, Roberts KW, Dufour MK, Whittemore B, Tambo M (2020). Effectiveness of reactive focal mass drug administration and reactive focal vector control to reduce malaria transmission in the low malaria-endemic setting of Namibia: a cluster-randomised controlled, open-label, two-by-two factorial design trial. Lancet.

[9] Lwin KM, Imwong M, Suangkanarat P, Jeeyapant A, Vihokhern B, Wongsaen K, Snounou G, Keereecharoen L, White NJ, Nosten F (2015). Elimination of plasmodium falciparum in an area of multi-drug resistance. Malar J.

[10] Adhikari B, Pell C, Phommasone K, Soundala X, Kommarasy P, Pongvongsa T, Henriques G, Day NPJ, Mayxay M, Cheah PY (2017). Elements of effective community engagement: lessons from a targeted malaria elimination study in Lao PDR (Laos). Glob Health Action.

[11] Tripura R, Peto TJ, Chea N, Chan D, Mukaka M, Sirithiranont P, Dhorda M, Promnarate C, Imwong M, von Seidlein L, Duanguppama J, Patumrat K, Huy R, Grobusch MP, Day NPJ, White NJ, Dondorp AM (2018). A controlled trial of mass drug administration to interrupt transmission of multidrug-resistant falciparum malaria in Cambodian villages. Clin Infect Dis.

[12] Peto TJ, Tripura R, Davoeung C, Nguon C, Nou S, Heng C, Kunthea P, Adhikari B, Lim R, James N, Pell C, Cheah PY (2018). Reflections on a community engagement strategy for mass antimalarial drug Administration in Cambodia. Am J Trop Med Hyg.

[13] Deng C, Wang Q, Zheng S, Zhou C, Gao Y, Guo J, Mliva AM, Oithik F, Bacar A, Attoumane R, Song J (2014). Mass drug Administration of Artemisinin-piperaquine on high malaria epidemic area. Trop Med Health.

[14] Ali AS, Thawer NG, Khatib B, Amier HH, Shija J, Msellem M, al-mafazy AW, Garimo IA, Mkali H, Ramsan MM, Kafuko JM, Paxton LA, Reithinger R, Ngondi JM (2017). Artemisinin combination therapy mass drug administration in a setting of low malaria endemicity: programmatic coverage and adherence during an observational study in Zanzibar. Malar J.

[15] Landier J, Parker DM, Thu AM, Lwin KM, Delmas G, Nosten FH, Andolina C, Aguas R, Ang SM, Aung EP, Baw NB, Be SA, B'Let S, Bluh H, Bonnington CA, Chaumeau V, Chirakiratinant M, Cho WC, Christensen P, Corbel V, Day NPJ, Dah SH, Delmas G, Dhorda M, Dondorp AM, Gaudart J, Gornsawun G, Haohankhunnatham W, Hla SK, Hsel SN, Htoo GN, Htoo SN, Imwong M, John S, Kajeechiwa L, Kereecharoen L, Kittiphanakun P, Kittitawee K, Konghahong K, Khin SD, Kyaw SW, Landier J, Ling C, Lwin KM, Lwin KSW, Ma NK'Y, Marie A, Maung C, Marta E, Minh MC, Miotto O, Moo PK, Moo KL, Moo M, Na NN, Nay M, Nosten FH, Nosten S, Nyo SN, Oh EKS, Oo PT, Oo TP, Parker DM, Paw ES, Phumiya C, Phyo AP, Pilaseng K, Proux S, Rakthinthong S, Ritwongsakul W, Salathibuphha K, Santirad A, Sawasdichai S, von Seidlein L, Shee PW, Shee PB, Tangseefa D, Thu AM, Thwin MM, Tun SW, Wanachaloemlep C, White LJ, White NJ, Wiladphaingern J, Win SN, Yee NL, Yuwapan D (2018). Effect of generalised access to early diagnosis and treatment and targeted mass drug administration on plasmodium falciparum malaria in eastern Myanmar: an observational study of a regional elimination programme. Lancet.

[16] von Seidlein L, Peto TJ, Landier J, Nguyen TN, Tripura R, Phommasone K, Pongvongsa T, Lwin KM, Keereecharoen L, Kajeechiwa L, Thwin MM, Parker DM, Wiladphaingern J, Nosten S, Proux S, Corbel V, Tuong-Vy N, Phuc-Nhi TL, Son DH, Huong-Thu PN, Tuyen NTK, Tien NT, Dong LT, Hue DV, Quang HH, Nguon C, Davoeung C, Rekol H, Adhikari B, Henriques G, Phongmany P, Suangkanarat P, Jeeyapant A, Vihokhern B, van der Pluijm RW, Lubell Y, White LJ, Aguas R, Promnarate C, Sirithiranont P, Malleret B, Rénia L, Onsjö C, Chan XH, Chalk J, Miotto O, Patumrat K, Chotivanich K, Hanboonkunupakarn B, Jittmala P, Kaehler N, Cheah PY, Pell C, Dhorda M, Imwong M, Snounou G, Mukaka M, Peerawaranun P, Lee SJ, Simpson JA, Pukrittayakamee S, Singhasivanon P, Grobusch MP, Cobelens F, Smithuis F, Newton PN, Thwaites GE, Day NPJ, Mayxay M, Hien TT, Nosten FH, Dondorp AM, White NJ (2019). The impact of targeted malaria elimination with mass drug administrations on falciparum malaria in Southeast Asia: a cluster randomised trial. PLoS Med.

[17] Luxemburger C, Thwai KL, White NJ, Webster HK, Kyle DE, Maelankirri L, Chongsuphajaisiddhi T, Nosten F (1996). The epidemiology of malaria in a Karen population on the western border of Thailand. Trans R Soc Trop Med Hyg.

[18] Imwong M, Nguyen TN, Tripura R, Peto TJ, Lee SJ, Lwin KM, Suangkanarat P, Jeeyapant A, Vihokhern B, Wongsaen K, van Hue D, Dong LT, Nguyen TU, Lubell Y, von Seidlein L, Dhorda M, Promnarate C, Snounou G, Malleret B, Rénia L, Keereecharoen L, Singhasivanon P, Sirithiranont P, Chalk J, Nguon C, Hien TT, Day N, White NJ, Dondorp A, Nosten F (2015). The epidemiology of subclinical malaria infections in South-East Asia: findings from cross-sectional surveys in Thailand-Myanmar border areas, Cambodia, and Vietnam. Malar J.

[19] Malaria Elimination Task Force. Activity report update may 2014 to December 2019. 2020.

[20] White MT, Conteh L, Cibulskis R, Ghani AC (2011). Costs and cost-effectiveness of malaria control interventions--a systematic review. Malar J.

[21] World Health Organisation. Review of delivery cost data on mass drug administration for malaria. 2015.

[22] Kyaw SS, Drake T, Thi A, Kyaw MP, Hlaing T, Smithuis FM, White LJ, Lubell Y (2016). Malaria community health workers in Myanmar: a cost analysis. Malar J.

[23] Galactionva K, Velarde M, Silumbe K, Miller J, McDonnell A, Aguas R, et al. Costing malaria interventions from pilots to elimination programmes. Malar J. 2020;19(1):332. 10.1186/s12936-020-03405-3 PMC7491157 32928227

[24] UN Habitat: Climate Profile, Myanmar. 2018.

[25] Jolliffe K: Ceasefires, Governance and Development: The Karen National Union in Times of Change. 2016.

[26] York Health Economics Consortium. Tornado Diagram [online]. 2016. https://yhec.co.uk/glossary/tornadodiagram/.

[27] Imwong M, Hanchana S, Malleret B, Renia L, Day NP, Dondorp A (2014). High-throughput ultrasensitive molecular techniques for quantifying low-density malaria parasitemias. J Clin Microbiol.

[28] Mu TT, Sein AA, Kyi TT, Min M, Aung NM, Anstey NM, Kyaw MP, Soe C, Kyi MM, Hanson J (2016). Malaria incidence in Myanmar 2005-2014: steady but fragile progress towards elimination. Malar J.

[29] World Health Organisation. Strategy for malaria elimination in the Greater Mekong Subregion (2015-2030). 2015.

[30] Shretta R, Avancena AL, Hatefi A (2016). The economics of malaria control and elimination: a systematic review. Malar J.

[31] World Health Organisation. A framework for malaria elimination. 2017.

[32] Landier J, Kajeechiwa L, Thwin MM, Parker DM, Chaumeau V, Wiladphaingern J, Imwong M, Miotto O, Patumrat K, Duanguppama J, Cerqueira D, Malleret B, Rénia L, Nosten S, von Seidlein L, Ling C, Proux S, Corbel V, Simpson JA, Dondorp AM, White NJ, Nosten FH (2017). Safety and effectiveness of mass drug administration to accelerate elimination of artemisinin-resistant falciparum malaria: a pilot trial in four villages of eastern Myanmar. Wellcome Open Res.

[33] Tedla M (2019). A focus on improving molecular diagnostic approaches to malaria control and elimination in low transmission settings: review. Parasite Epidemiol Control.

[34] Yukich JO, Scott C, Silumbe K, Larson BA, Bennett A, Finn TP, et al. Cost-effectiveness of focal mass drug administration and mass drug administration with Dihydroartemisinin–Piperaquine for malaria prevention in Southern Province, Zambia: Results of a Community-Randomized Controlled Trial. 2020. 10.4269/ajtmh.19-0661 PMC7416981 32618249

[35] Hamilton WL, Amato R, van der Pluijm RW, Jacob CG, Quang HH, Thuy-Nhien NT, Hien TT, Hongvanthong B, Chindavongsa K, Mayxay M, Huy R, Leang R, Huch C, Dysoley L, Amaratunga C, Suon S, Fairhurst RM, Tripura R, Peto TJ, Sovann Y, Jittamala P, Hanboonkunupakarn B, Pukrittayakamee S, Chau NH, Imwong M, Dhorda M, Vongpromek R, Chan XHS, Maude RJ, Pearson RD, Nguyen T, Rockett K, Drury E, Gonçalves S, White NJ, Day NP, Kwiatkowski DP, Dondorp AM, Miotto O (2019). Evolution and expansion of multidrug-resistant malaria in Southeast Asia: a genomic epidemiology study. Lancet Infect Dis.

[36] Parker DM, Landier J, Thu AM, Lwin KM, Delmas G, Nosten FH, The Malaria Elimination Task Force Group (2017). Scale up of a plasmodium falciparum elimination program and surveillance system in Kayin State, Myanmar. Wellcome Open Res.

[37] Gao B, Saralamba S, Lubell Y, White LJ, Dondorp AM, Aguas R. Determinants of MDA impact and designing MDAs towards malaria elimination. Elife*.* 2020;9. 10.7554/eLife.51773. 10.7554/eLife.51773 PMC7185997 32293559

