# Supplementary material for: Estimating the programmatic cost of targeted mass drug administration for malaria in Myanmar
Source: BMC Public Health. 2021 Apr 29;21:826. doi: 10.1186/s12889-021-10842-5 (PMC8082869; doi:10.1186/s12889-021-10842-5)
Supplement: Supplementary file 1 — Additional file 1. The interactive costing tool. This file contains the features of malaria mass intervention costing tool. [file 12889_2021_10842_MOESM1_ESM.docx]

Developing the interactive costing tool

We developed an interactive malaria mass intervention costing tool, using the R programming language, to estimate the full costs of implementing malaria mass interventions. The tool is available via the Mathematical and Economic Modelling group website <https://moru.shinyapps.io/Mass-Malaria-Interventions-Costing-Tool/>.

Supplementary Figure 1 shows a screenshot of the mass malaria intervention costing tool. The main aim in developing this tool was to estimate the programmatic costs of mass malaria interventions intended to eliminate *P*. *falciparum* malaria in the elimination setting. The web application was developed based on unit costs estimated from a pilot MDA project implemented in Kayin State, Myanmar. Users can evaluate the costs of different malaria mass interventions for their intended setting, or scale up the costs of an existing MDA programme, by moving the slider bars in the application. The costs of programmes change as users change the value of variables on the slider bars. Supplementary Table 1 shows the parameters and the values used to estimate the programmatic cost of targeted MDA in Kayin State.


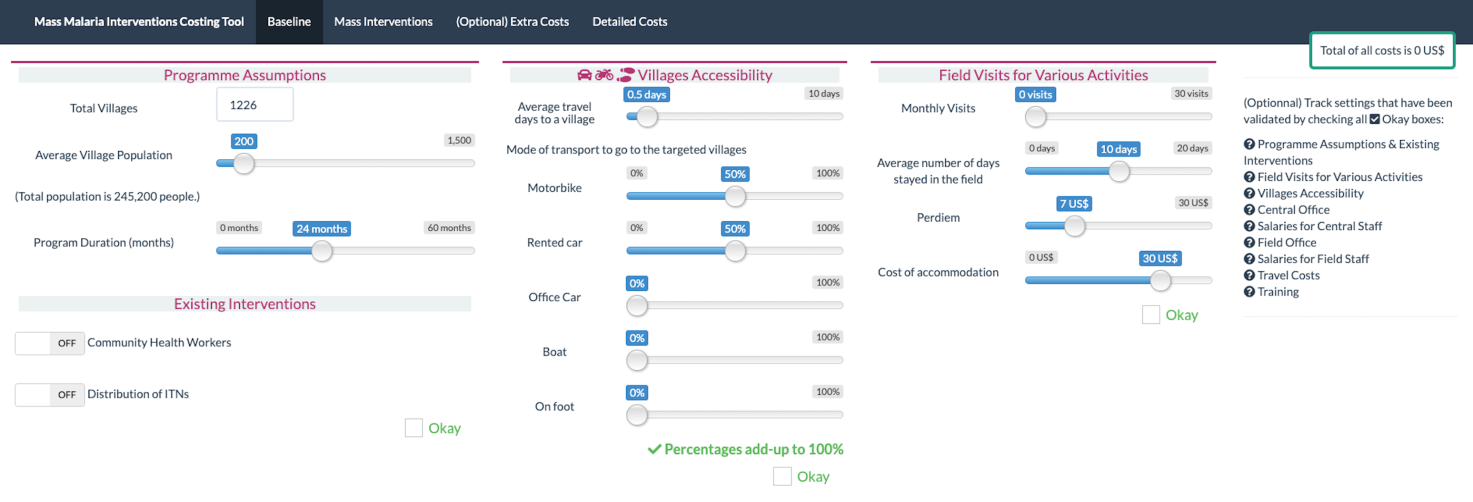


Supplementary Figure 1 A screenshot of the mass malaria intervention costing tool

In this costing tool, the users can estimate the existing malaria interventions such as community health workers programme and distribution of long-lasting insecticide-treated bed-nets. The user can predict the cost of malaria elimination using malaria mass interventions. If the pre-assigned resources are not enough to predict the budget, this tool provides a component that the users are allowed to add the resources as an extra/optional. The costs of the extra/optional resources are included in the total estimated cost. Summary of the total costs is displayed in a costing table and a bar graph by activity or resources used in a program.

Outputs of the costing model

Outputs are displayed in a table and a bar graph by activity or resource used in the interventions. Although local currencies are used in-country, the preparation and submission of budgets to funder organisations are generally made using United States dollars (US$). Therefore, the outputs of the tool are given in US$.

There are four tabs in the malaria mass intervention costing tool. When a user clicks the link provided above, an introduction page appears and briefly explains the purpose behind the development of the malaria mass intervention costing model and how to use it. The contact details of the tool’s developers are available in case the user would like to know more about the costing model or provide comments or suggestions.

Baseline

The second tab collects baseline information regarding the setting and the unit cost of resources to estimate the cost of an intervention. If the setting has existing malaria interventions, for example community health workers (CHWs) or the distribution of insecticide-treated bed-nets (ITNs), the tool can also estimate their maintenance costs. Information regarding village accessibility and field visits to perform various activities was also collected, as were the unit costs and salaries of field and management staff.

Mass interventions

The third tab is the mass intervention tab. Community engagement forms an essential foundation in the preparation for any mass intervention. The community engagement can be clicked if the user wants to add any costs of community engagement to their budget. The user can also adjust information regarding community engagement under the community engagement tab.

Mass interventions include (i) mass drug administration, (ii) mass screening and treatment, (iii) mass vaccination, (iv) mass drug administration plus mass vaccination, and (v) mass screening and treatment plus mass vaccination. The user can choose one malaria intervention for their intended setting by selecting the appropriate radio button.

A useful feature incorporated into this costing tool is a targeting strategy option. If a user would like to target a proportion of villages that have higher numbers of malaria cases in their communities, they can select one of three targeting strategies. These different targeting strategies that can be used to identify hotspot villages are (i) mass blood screening using a quantitative uPCR method, (ii) reviewing monthly malaria reports from CHWs, and (iii) providing antimalarial drugs to all villages regardless of their malaria burden (no targeting).

Optional (extra costs)

All ingredients consumed in the pilot MDA projects are included in the interactive malaria mass intervention costing model. However, if a user would like to add any resources to their budget that are not included in the built-in ingredients, the tool allows them to add additional components. The user can fill in the name of these ingredients, their unit costs, and the quantity required in their intended programme. The values of these items will then be calculated and added to the total costs. Users can add up to a maximum of six items in the development of their budget.

Exporting data

The user can save the values of the parameters they used in the budget estimation for future reference. They can also export a summary of the cost of their programme roll-out or future project by clicking the download button in the last tab (Detailed costs) of the costing tool. This will create a comma-separated values (CSV) file that can be saved.

Supplementary Table 1 The parameters used in the costing model and the value of the parameters used to estimate the costs of *P*. *falciparum* malaria elimination in Kayin State, Myanmar

| **Parameter** | **Value** | **Unit** |
| --- | --- | --- |
| Programme assumptions |  |  |
| Total number of villages | 1 226 | Village |
| Average village population | 200 | People |
| Project duration | 12 | Month |
|  |  |  |
| Village Accessibility |  |  |
| Motorbike | 50 | Percentage |
| Rented car | 50 | Percentage |
| Salaries for central staff |  |  |
| General director | 8 000 | US$ |
|  |  |  |
| Salaries for field staff |  |  |
| Team leader | 1 000 | US$ |
| Programme manager’s assistant | 500 | US$ |
| Lab staff | 500 | US$ |
| Community health worker | 50 | US$ |
| Logistics | 200 | US$ |
|  |  |  |
| Travel Costs |  |  |
| Travel cost per village via motorbike | 25 | US$ |
| Travel cost per village via rented car | 100 | US$ |
|  |  |  |
| Parameters for community engagement (CE) |  |  |
| Number of days spent in a village for CE | 1 | Day |
| Staff involved in CE |  |  |
| Team leader | 0 | Person |
| Program manager assistant | 1 | Person |
| Helpers/CHWs/ volunteers | 2 | Person |
|  |  |  |
| Equipment |  |  |
| Equipment cost per village for one community engagement activity | 0 | US$ |
|  |  |  |
| Consumables |  |  |
| Consumables cost per village for community engagement | 18 | US$ |
|  |  |  |
| Incentives |  |  |
| Refreshment costs per village during community engagement activity | 19 | US$ |
| Costs of community incentives | 4 828 | US$ |
| Number of villages provide with community incentives | 3 | Village |
| Out of all villages, the percentage of villages visited for community engagement | 22 | Percentage |
|  |  |  |
| Training |  |  |
| Number of training sessions for CE activities | 7 | Session |
| Number of participants for CE training | 10 | Person |
| Number of trainers for CE training | 2 | Person |
| Duration of a training session for CE activities | 2 | Day |
|  |  |  |
| Parameters specific to uPCR |  |  |
| Percentage of villages surveyed to identify villages for targeted MDA | 22 | Percentage |
|  |  |  |
| Personnel |  |  |
| Number of days spent in a village for targeted MDA activity to identify targeted MDA villages | 1 | Day |
| Team leader/program manager/supervisor | 0 | Person |
| Program manager’s assistant/logistics assistant | 2 | Person |
| Laboratory staff | 0 | Person |
| Helper/CHW | 2 | Person |
|  |  |  |
| Incentives |  |  |
| The incentive for a participant to donate blood | 1 | US$ |
|  |  |  |
| Equipment |  |  |
| The equipment cost per village for mass blood survey activities | 5 | US$ |
|  |  |  |
| Consumables |  |  |
| Consumables costs for uPCR tests | 1 | US$ |
| uPCR analysis cost per test | 25 | US$ |
|  |  |  |
| Training |  |  |
| Number of training sessions for the uPCR method | 8 | Session |
| Number of participants for uPCR training | 15 | Person |
| Number of trainers for uPCR training | 2 | Person |
| Duration of a training session for uPCR | 2 | Day |
|  |  |  |
| Monitoring and supervision |  |  |
| Number of trips for monitoring uPCR activity | 1 | Trip |
| Number of days spent monitoring uPCR activity | 5 | Day |
|  |  |  |
| Parameters for mass drug administration |  |  |
| Percentage of villages offered MDA activity | 21 | Percentage |
| Number of MDA rounds in a year | 3 | Round |
| Average population coverage for MDA in a round | 85 | Percentage |
|  |  |  |
| Personnel |  |  |
| Number of days spent in a village for MDA activity | 7 | Days |
|  |  |  |
| Incentives |  |  |
| The incentive for one participant in a round of MDA activity (US$) | 1 | US$ |
|  |  |  |
| Travel |  |  |
| Number of a trips to a village for MDA activity (1 round) | 1 | Trip |
| Number of trips for car rental during MDA activity (1 round) | 15 | Trips |
|  |  |  |
| Equipment |  |  |
| Equipment cost per village for MDA activity | 25 | US$ |
|  |  |  |
| Consumables |  |  |
| Consumables cost per village for targeted MDA activity | 20 | US$ |
| Cost of DHA+ PQP, blister pack child | 0·93 | US$ |
| Cost of DHA + PQP, blister pack youth | 1·46 | US$ |
| Cost of DHA + PQP, blister pack adult | 1·98 | US$ |
| Cost of primaquine base 7.5 mg tablet | 0·01 | US$ |
|  |  |  |
| Cost of medicine for the treatment of side-effects | 39 | US$ |
| Antimalarial drug wastage | 5 | Percentage |
|  |  |  |
| Training |  |  |
| Number of training sessions for targeted MDA | 8 | Session |
| Number of participants for targeted MDA training | 10 | Person |
| Number of trainers for targeted MDA training | 3 | Person |
| Duration of a training session for targeted MDA | 3 | Day |
|  |  |  |
| Monitoring and supervision |  |  |
| Number of monitoring trips for targeted MDA (1 round) | 1 | Trip |
| Duration of monitoring trips for targeted MDA (1 round) | 10 | Day |

CHW, community health worker; MDA, mass drug administration; uPCR, ultrasensitive polymerase chain reaction; DHA, dihydroartemisinin; PQP, piperaquine phosphate
